# Association between the type of provider and Cesarean section delivery in India: A socioeconomic analysis of the National Family Health Surveys 1999, 2006, 2016

**DOI:** 10.1371/journal.pone.0248283

**Published:** 2021-03-08

**Authors:** Hwa-Young Lee, Rockli Kim, Juhwan Oh, S. V. Subramanian

**Affiliations:** 1 Department of Global Health and Population, Harvard T.H. Chan School of Public Health, Boston, Massachusetts, United States of America; 2 Institute of Convergence Science (ICONS) Convergence Science Academy, Yonsei University, Seoul, Korea; 3 Division of Health Policy and Management, College of Health Sciences, Korea University, Seoul, Korea; 4 Department of Public Health Sciences, Interdisciplinary Program in Precision Public Health, Graduate School of Korea University, Seoul, Korea; 5 Harvard Center for Population & Development Studies, Cambridge, Massachusetts, United States of America; 6 Department of Medicine, Seoul National University College of Medicine, Seoul, Korea; 7 Department of Social and Behavioral Sciences, Harvard T.H. Chan School of Public Health, Boston, Massachusetts, United States of America; University of Mississippi Medical Center, UNITED STATES

## Abstract

**Background:**

Prevalence of Cesarean section (C-section) is unequally distributed. Since both extremely low and high levels of C-section can not only cause adverse birth outcomes but also impose a double burden of inefficiency within maternal health care, it is important to monitor the dynamics of key factors associated with the use of C-section.

**Objectives:**

To examine the association between type of provider and C-section in India in three-time points: 1999, 2006, and 2016, and also to assess whether this association differed across maternal education and wealth level.

**Methods:**

Data were from three waves of cross-sectional and nationally representative Indian National Health Family Survey: Wave II (1999), III (2006), and IV (2016). Target population is women aged 15 and 49 who had an institutional delivery for the most recent live birth during the three or five years preceding the survey (depending on the survey round). Multivariate logistic regression models adjusting for state cluster effect were performed to determine the association between the type of providers and C-section. Differential association between the type of providers and C-section by maternal education and wealth level was examined by stratified analyses.

**Results:**

The prevalence of C-section among institutional delivery increased from 20.5% in 1999 to 24.8% in 2006 while it declined to 19.4% in 2016. The positive association between private providers and C-section became stronger over the study period (Odds Ratio (OR) = 1.39, 95% Confidence Interval (CI) 1.18–1.64 in 1999, OR = 3.71 95% CI 2.93–4.70 in 2016). The association was consistently significant across all states in 2016. The gap in C-section between public and private providers was greater among less-educated and poorer women. The ORs gradually increased from the poorest to the richest quintiles, and also from the least educated group (no formal education) to the most educated group (college graduate or above)

**Conclusions:**

Our results suggest that disparity in C-section between private and public providers has increased over the last 15 years and was higher in lower SES women. The behavior of providers needs to be closely monitored to ensure that C-section is performed only when medically justified.

## Introduction

Availability and quality of comprehensive obstetric care is the key to reduce preventable maternal mortality [1]. Cesarean section (C-section), an essential component of obstetric care, can save maternal and fetal lives by preventing complications for labor when medically justified [2]. On the other hand, medically unnecessary C-section does not benefit women and their newborns, and may even increase short-term and long-term health risks [3].

Prevalence of C-section has increased in all regions of the world, except Sub-Saharan Africa, with the global average increasing from 6.7% in 1990 to 19.1% in 2014 [4]. However, in some of the poorer countries, or among certain subgroups within countries, it remains extremely low, indicating the possibility of the lack of access to adequate obstetric care. Although the international healthcare community has proposed an ideal rate for C-section to be between 10% and 15% [5], the optimal rate of C-section in each context is difficult to identify because of challenges in ascertaining the rate of true indications of medical needs at the population level.

India has also experienced a fast-growing C-section rate with substantial within-country inequality. The national prevalence of C-section was 17.2% in 2016, but state prevalence varied greatly from 6.2% in Bihar to 57.7% in Telangana [6]. Since both extremely low and high levels of C-section can not only cause adverse birth outcomes but also impose a double burden of inefficiency within maternal health care [7, 8], it is important to understand and monitor the dynamics of key factors associated with the use of C-section.

Previous studies have identified various factors associated with the utilization of C-section, among which the patient’s socioeconomic status (SES) and type of provider were found to be key contributors [9]. It was demonstrated that women’s higher SES and private hospital are more strongly associated with C-section delivery compared to their counterparts in Low and Middle-Income Countries (LMICs) [7, 9–12]. Cavallaro et al. reported an increasing (unadjusted) gap in prevalence of C-section among wealth quintiles over the years in 26 Southern Asian or Sub-Saharan African countries [7]. On the other hand, no study to date has examined the time trend of public-private difference in C-section (whether adjusted or unadjusted).

Since the final indication to perform C-section is determined by the surgeon–whether it is initiated from the patient’s request or the doctor’s own judgment–the nature of providers in relation to C-section deserves more attention. It is highly likely that the influence of provider factors on C-section is not static over time, given the concern about the privatization of health care and over-medicalization of maternal care in India [13]. Also, the choice of C-section is a complex social process, influenced by a combination of various factors including clinical status, family and social factors, availability of technology, and women’s perception toward the C-section, etc [14]. Therefore, provider characteristics are not likely to act independently of other factors in the choice of C-section.

Although health system depends on state policy, reproductive services including antenatal care and delivery are provided mostly for free or at nominal cost in public health centers in India [15]. On the other hand, patients have to pay a significant amount of out-of-pocket (OOP) cost in private facilities because the governments neither regulate the service-fee charged by private providers nor do they support the cost for service utilization in the private facilities except for some empanelled ones in special government schemes such as Ayushman Bharat or Janani Suraksha Yojana (JSY) that aim to ensure accessibility to the health service of the poor and the vulnerable [16]. Therefore, there is a big gap in OOP expenditure for C-section between public and private facilities [15, 16]. Given this, it is expected that, above all factors, women’s economic status is the most likely to interact with provider type in the utilization of C-section. Also, women’s education level affects women’s knowledge of and perception toward the risk and benefit of C-section, and therefore, influences the capacity to make the right decision on the utilization of the C-section [17].

To date, most of the evidence on public-private provider difference in the utilization of C-section in India was based on a single time point, covered a limited geographic area, or focused the association between the type of provider and C-section without considering the role of other factors in the association [10, 11, 18–22]. To address these gaps in the literature, this study aimed to 1) investigate a long-term trend in the associations between type of provider (public vs. private) and C-section based on nationally representative, repeated cross-sectional surveys from 1999, 2006, and 2016 and 2) examine interactions between type of provider and maternal wealth and education level in the utilization of C-section based on the latest survey data.

## Materials and methods

### Data

The data used in the present study was taken from three waves of the National Health Family Survey (NFHS) II (1998–1999), III (2005–2006), and IV (2015–2016) [6, 23, 24]. NFHS is nationally representative and is conducted by the International Institute for Population Sciences with the aim to collect information on a range of maternal and child health through interviews with women aged 15–49 years [25].

A stratified two-stage sample design in rural areas and a three-stage design in urban areas were adopted for NFHS II and III, while a stratified two-stage sampling was used for both rural and urban areas in NHFS IV. For NFHS II and III, the primary sampling units (PSU) in rural areas, which corresponded to villages, were selected with probability proportional to population size (PPS) in the first stage, followed by a random selection of households within each PSU at the second stage. In urban areas, PSUs, which corresponded to the wards, were also selected with PPS sampling in the first stage and then one census enumeration block (CEB) was randomly selected from each sample ward at the second stage. Finally, households were randomly selected within each selected CEB. For NFHS IV, village and CEB served as PSU in rural and urban areas, respectively [6, 23, 24].

### Variables

The outcome variable was C-section of the last birth, which was identified by self-report from the mother and was measured in a binary response: yes or no. The primary predictor was the type of provider where mothers gave birth, classified as public (including government/municipality hospital, government dispensary, urban health center or post, an urban family welfare center, community health center, block primary health center, sub-center) or private (including private hospital/maternity home/clinic, non-government organization, and trust hospital/clinic). Maternal education was categorized as no education, primary graduate or less, secondary graduate or less, and college or above. Household wealth was presented in quintiles generated from a composite wealth index, which was estimated from a set of multiple household assets and characteristics such as dwelling’s construction material, source of drinking water, sanitation facility, cooking fuels, and so on, using principal component analysis [26, 27].

Covariates were selected a priori based on a literature review. Demographic factors included maternal age at pregnancy (<20 vs. 20 ≦age<30, 30≦age<35, and ≧35) [28], birth order (1^st^ vs. 2^nd^, 3^rd^, and ≥ 4^th^) [29], and baby gender (male vs. female) [30]. Covariates related to medical need included baby size self-reported by mothers (average vs. very large, larger than average, smaller than average, and very small), multiple gestations (singleton vs. multiple) [31], women’s stature (normal vs. short defined as <155cm), and women’s body mass index (BMI) (<18.5, ≧18.5 & <25, ≧25&<30, and 30 ≦) [32]. Indicators for smoking and drinking alcohol were operationalized as binary variables [33, 34]. Delivery complication defined as having experienced one of the three symptoms during delivery (breech presentation, prolonged labor, and excessive bleeding) and history of terminated pregnancy from miscarriage, abortion, or stillbirth (no vs. yes) were also included [35]. SES covariates, other than education and household wealth level, included the type of residence (rural vs. urban), and caste (scheduled tribe (ST) vs. scheduled caste (SC), other backward class, and others) [36]. Castes in India, a part of a complex system of social stratification where STs are more isolated physically and socially and disadvantaged compared to SCs or other castes [37], was found to be significantly associated with poor access to the C-section in previous studies [36]. Institutional factors included a history of more than four antenatal care visits (no vs. yes) and insurance coverage (no vs. yes) [38]. Information on the history of delivery complications and terminated pregnancy were only available in NFHS IV and information on health insurance was provided in NFHS III and IV only.

### Statistical analysis

Our study sample was defined as the most recent live birth in the three years preceding NFHS II and five years preceding NFHS III and IV among institutional deliveries [25]. First, we performed descriptive statistics of the study sample and presented the crude prevalence of C-section by the independent variables in 1999, 2006, and 2016. While C-section prevalence in previous studies was estimated using the total number of births including home delivery as the denominator [20], we considered only institutional delivery (35.0%, 45.4%, and 75.5% among all births in 1999, 2006, and 2016 respectively) because C-section can only be performed in facility. Unadjusted and adjusted logistic regressions were conducted to examine the associations between type of provider and C-section as well as to compare the strength of associations before and after adjusting for a comprehensive set of covariates. We tried parsimonious models where we restricted to covariates that were statistically significantly associated with C-section. The coefficients for the type of provider hardly changed, and Akaike information criterion (AIC) and Bayesian information criterion (BIC) increased a little bit in all three years in parsimonious models (Results shown in S1 Table). Therefore, we kept all the covariates that were selected a priori by a review of previous studies for the main analyses. Regression analyses were run separately by survey year with the same set of covariates. State-stratified analyses based on NFHS IV were additionally conducted to examine the consistency of the findings across the states. Finally, we examined whether there is heterogeneity in estimates of associations between type of provider and C-section by stratifying the sample of NFHS IV by the maternal education and wealth level.

Logistic regression assumes that responses on each observation are independent of each other. However, when a cluster effect is anticipated, the responses may be correlated with each other within certain strata which leads to an underestimation of standard error. The distribution of important health outcomes such as C-section may be clustered at the state level in India where health is a subject of state government. Therefore, we introduced the state cluster effect (u_i_) to the model as a normally distributed random variable with zero mean and constant variance, given by the model as follows:

$$logit\;\left(\frac{p_{ij}}{1-p_{ij}}\right)=\alpha +{\beta x}_{i}+u_{j}$$

$$\mathrm{where}\;u_{i}∼N\;(0,\;\sigma_{u}^{2})$$

x_i_ is a fixed effect for all the independent variables and u_j_ is a state random effect. Huber’s method of adjustment was used to adjust for the bias, which is available in the statistical package STATA [39]. Stata 14.0 software was used for all estimates.

### Ethics statement

Ethics approval from our respective institutions was not required because our study was limited to the publicly available NFHS dataset that contained no personally identifiable information.

## Results

The analytic sample was composed of 10,247, 15,412, and 133,627 births in NFHS II, III, and IV respectively after excluding observations with missing information on each of the variables (S1 Fig). The characteristics of the analytic sample and the original sample were similar (S2 Table).

Table 1 presents the descriptive statistics of the analytic samples. The proportion of women living in rural areas and from the poorest and poor households was higher in NFHS IV compared to in NFHS II. The prevalence of C-section increased from 20.5% in 1999 to 24.8% in 2006 but decreased to 19.4% in 2016.

**Table 1:** Descriptive statistics of analytical samples.

Variable	Frequency (%)						Prevalence of C-sec		
	NFHS II (1999)		NFHS III (2006)		NFHS IV (2016)		NFHS II (1999)	NFHS III (2006)	NFHS IV (2016)
Age at pregnancy									
<20	3,546	(15.0)	1,635	(10.6)	10,259	(7.7)	7.5	19.7	16.0
≧ 20 and <30	6,701	(70.7)	11,029	(71.6)	99,389	(74.4)	22.3	24.1	19.0
≧ 30 and <35	1,102	(10.8)	2,087	(13.5)	17,330	(13.0)	23.8	30.6	22.6
≧ 35	365	(3.6)	661	(4.3)	6,649	(5.0)	23.0	30.9	21.8
Birth order									
First	4,490	(43.8)	6,074	(39.4)	49,188	(36.8)	25.5	30.1	25.5
Second	3,046	(29.7)	5,487	(35.6)	46,258	(34.6)	20.8	26.1	20.9
Third	1,433	(14.0)	2,122	(13.8)	21,268	(15.9)	14.7	17.7	12.3
Higher than four	1,278	(12.5)	1,729	(11.2)	16,977	(12.7)	9.2	10.5	6.5
Gender of baby									
Male	5,422	(52.9)	8,417	(54.6)	72,980	(54.6)	21.6	24.7	19.3
Female	4,825	(47.1)	6,995	(45.4)	60,647	(45.4)	19.3	24.8	19.5
Baby size									
Very large	0	(0)	669	(4.3)	7,481	(5.6)	0	31.5	24.9
Larger than average	1,830	(17.9)	3,310	(21.5)	17,427	(13.0)	28.1	25.6	22.8
Average	6,132	(59.8)	8,571	(55.6)	93,597	(70.0)	18.8	24.3	18.4
Smaller than average	1,856	(18.1)	2,080	(13.5)	11,667	(8.7)	18.3	22.9	18.8
Very small	429	(4.2)	782	(5.1)	3,455	(2.6)	22.6	25.6	19.7
Plurality of pregnancy									
Singleton	10,099	(98.6)	15,250	(98.9)	132,479	(99.1)	20.4	24.6	19.2
Twin or triplet	158	(1.5)	162	(1.1)	1,148	(0.9)	28.5	37.7	38.7
Mother’s height									
Not short (height≧155cm)	2,980	(29.1)	5,102	(33.1)	39,578	(29.6)	19.9	25.3	20.5
Short (height<155cm)	7,267	(70.9)	10,310	(66.9)	94,049	(70.4)	20.8	24.5	18.9
BMI									
>30	190	(1.9)	542	(3.5)	4,529	(3.4)	44.2	49.1	46.4
≧25 and <30	934	(9.1)	2,205	(14.3)	17,556	(13.1)	32.3	37.2	33.9
≧18.5 and <30	6,717	(65.6)	9,740	(63.2)	88,987	(66.6)	20.1	23.0	17.1
<18.5	2,406	(23.5)	2,925	(19.0)	22,555	(16.9)	15.3	16.8	11.9
Smoking									
No	10,143	(99.0)	14,053	(91.2)	122,452	(91.6)	20.5	25.5	19.9
Yes	104	(1.0)	1,359	(8.8)	11,175	(8.4)	20.2	17.6	13.6
Alcohol									
No	10,125	(98.8)	15,159	(98.4)	131,519	(98.4)	20.6	24.7	19.5
Yes	122	(1.2)	253	(1.6)	2,108	(1.6)	17.2	28.1	15.3
Complication									
No	n.a	n.a	n.a	n.a	63,242	(47.3)	n.a	n.a	18.5
Yes	n.a	n.a	n.a	n.a	70,385	(52.7)	n.a	n.a	20.2
Terminated pregnancy									
No	n.a	n.a	n.a	n.a	119,124	(89.1)	n.a	n.a	18.9
Yes	n.a	n.a	n.a	n.a	11,063	(10.9)	n.a	n.a	23.7
Maternal education									
No education	2,274	(22.2)	2,573	(16.7)	30,042	(22.5)	14.1	15.2	8.9
Primary graduate or less	1,690	(16.5)	1,845	(12.0)	17,119	(12.8)	16.1	18.4	13.0
Secondary graduate or less	4,129	(40.3)	8,316	(54.0)	68,717	(51.4)	20.7	24.8	21.3
Collage or above	2,154	(21.0)	2,678	(17.4)	17,749	(13.3)	30.5	38.4	36.1
Type of residence									
Urban	5,068	(49.5)	8,892	(57.7)	38,129	(28.5)	22.7	27.3	28.6
Rural	5,179	(50.5)	6,520	(42.3)	95,498	(71.5)	18.4	21.3	15.7
Caste									
Scheduled caste	1,470	(14.3)	2,337	(15.2)	26,078	(19.5)	16.6	21.5	16.9
Scheduled tribe	916	(8.9)	1,500	(9.7)	22,847	(17.1)	14.8	15.9	12.6
Other backward class	2,978	(29.1)	5,144	(33.4)	57,082	(42.7)	21.9	23.9	19.6
Others	4,883	(47.7)	6,431	41.7)	27,620	(20.7)	21.9	28.7	27.0
Wealth level									
1^st^ quintile(poorest)	522	(5.1)	694	(4.5)	25,109	(18.8)	17.4	12.8	6.9
2^nd^ quintile	915	(8.9)	1,337	(8.7)	28,192	(21.1)	12.3	16.3	11.7
3^rd^ quintile	1,721	(16.8)	2,633	(17.1)	28,453	(21.3)	14.4	17.9	19.1
4^th^ quintile	3,115	(30.4)	4,316	(28.0)	26,895	(20.1)	18.9	22.7	26.2
5^th^ quintile(richest)	3,974	(38.8)	6,432	(41.7)	24,978	(18.7)	26.7	32.0	33.8
Insurance									
Covered	n.a	n.a	1,110	(7.2)	20,456	(15.3)	n.a	32.3	22.5
Not covered	n.a	n.a	14,302	(92.8)	113,171	(84.7)	n.a	24.2	18.9
Type of provider									
Public	5,593	(54.6)	7,972	(51.7)	94,363	(70.6)	16.6	18.7	11.1
Private	4,654	(45.4)	7,440	(48.3)	39,264	(29.4)	25.2	31.3	39.4
Antenatal care ≧ 4 times									
No	3,546	(34.6)	4,088	(26.5)	60,924	(45.6)	12.8	15.1	12.3
Yes	6,701	(65.4)	11,324	(73.5)	72,703	(54.4)	24.6	28.3	25.4
Total	10,247		15,412		133,627				
Prevalence							20.5	24.8	19.4

Since seven union territories were newly added in NFHS IV, characteristics of the 29 states after excluding these seven union territories were compared to the analytic sample of 36 states. Characteristics of the study population and the prevalence of C-section between the two samples were similar (S3 Table).

**Fig 1:**
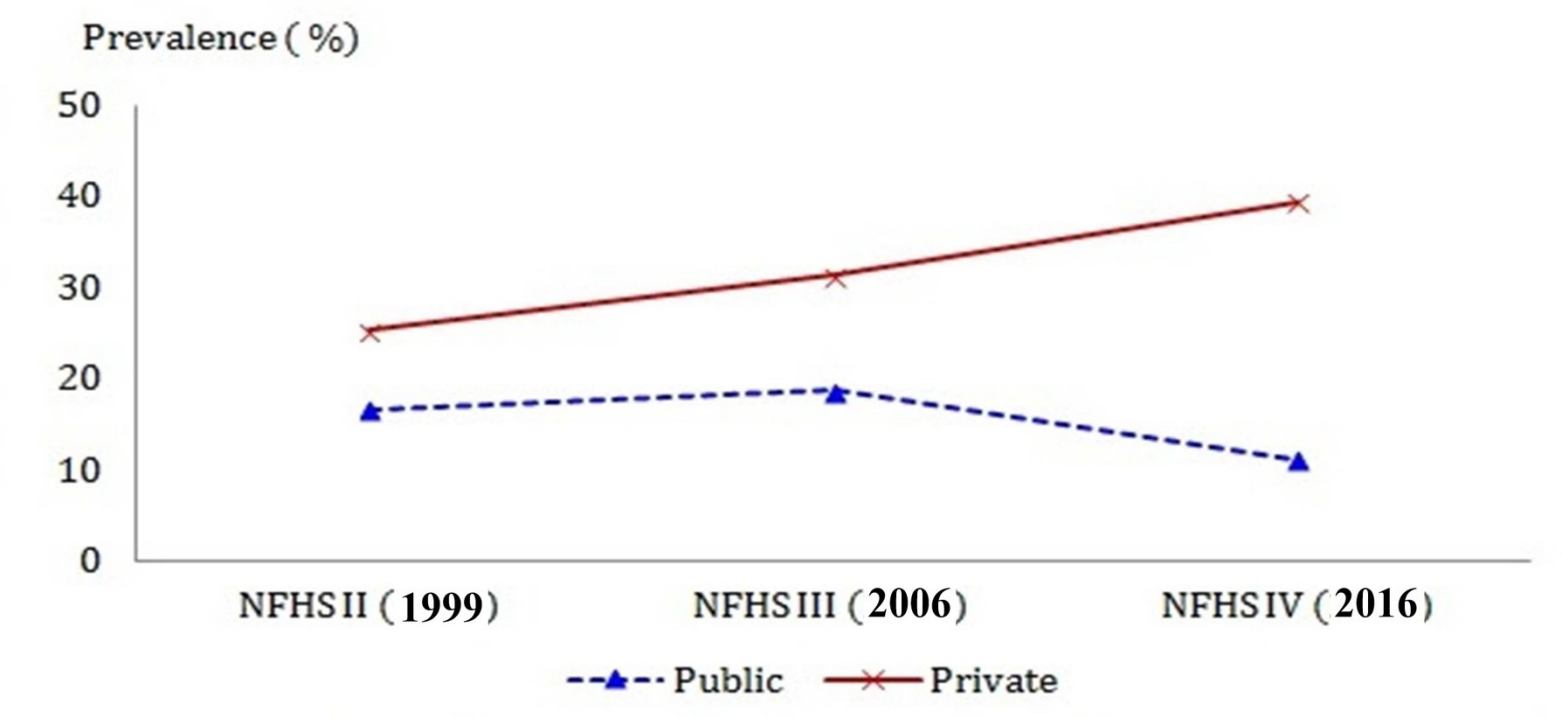
Prevalence of C-section by the type of provider in NFHS II, III, and IV.

The prevalence of C-section by private providers increased throughout the study period, i.e. 25.2% in 1999, 31.3% in 2006, and 39.4% in 2016, while the prevalence of C-section by public providers declined between 2006 and 2016 from 18.7% to 11.1% after a slight rise between 1999 and 2006 from 16.6% to 18.7%, resulting in a rate ratio of 3.5 in 2016. (Table 1 and Fig 1).

The unadjusted model indicates that the gap in the C-section rate between the public and private providers continuously widened. Especially, the increase in the gap was much larger during the later part of the period (Odds ratio (OR) 1.69, 1.98, and 5.20, 95% CI 1.46–1.96, 1.71–2.28, and 4.01–6.74 in 1999, 2006, and 2016, respectively). The strength of associations from a fully-adjusted model was attenuated compared to unadjusted associations but remained strongly significant throughout the study period (OR: 1.39, 1.59, and 3.71, 95% CI: 1.18–1.64, 1.40–1.81, and 2.93–4.70 in 1999, 2006, and 2016, respectively) (Table 2).

**Table 2:** The trend in adjusted and unadjusted associations between type of provider and C-section in 1999, 2006, and 2016.

Place of delivery		Unadjusted				Adjusted			
		OR		95% CI		OR		95% CI	
Public(ref)									
Private	NFHS II (1999)	1.69	***	1.46 -	1.96	1.39	***	1.18 -	1.64
	NFHS III (2006)	1.98	***	1.71 -	2.28	1.59	***	1.40 -	1.81
	NFHS IV (2016)	5.20	***	4.01 -	6.74	3.71	***	2.93 -	4.70

(All three waves were adjusted for age at pregnancy, birth order, baby gender, baby size, a plurality of pregnancy, short stature, BMI, smoking, drinking alcohol, maternal education, type of residence, caste, wealth level, ANC more than 4 times. History of delivery complication and terminated pregnancy, and health insurance status were excluded from the adjusted model for consistency across three waves) (***: p<0.001, **: p<0.01, *: p<0.05).

In a state-stratified analysis based on NFHS IV, a strong association between type of provider and C-section was observed across all states except for the Dadra and Nagar Haveli (S4 Table).

**Fig 2:**
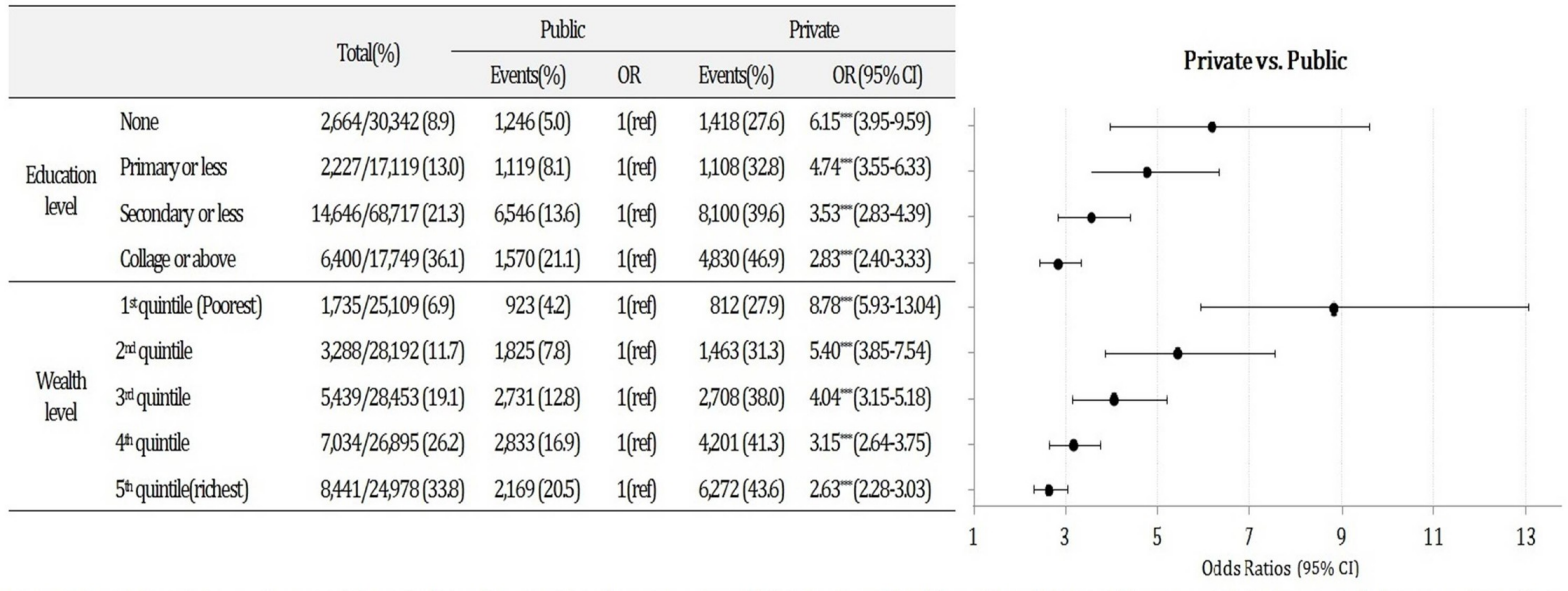
Associations between the type of provider and C-section by maternal education and wealth level in NFHS IV.

In 2016, there was a significant negative interaction between the type of provider and maternal education and wealth level on the C-section (results are not shown). To facilitate the interpretation of these effects, we performed the stratified analyses by education and wealth level (Fig 2). C-section was more likely to be performed among private providers compared to the public providers across all education and wealth strata. Notably, the gap was greater among women with lower education and from poorer households. The OR of C-section by private providers versus public providers for the women without formal education was 6.15 (95% CI 3.95–9.59) while the gap was much smaller among women with the highest education level (OR 2.83, 95% CI 2.40–3.33). Similarly, women from the poorest household were 8.78 times more likely to have C-section delivery by the private providers compared to by the public providers (95% CI 5.93–13.04) while the gap was much smaller at higher wealth levels. (OR 5.40, 4.04, 3.15, and 2.63, 95% CI 3.85–7.54, 3.15–5.18, 2.64–3.75, 2.28–3.03 for the 2^nd^, 3^rd^, 4^th^, and 5^th^ quintiles).

As for other covariates, older pregnancy age, higher birth order, the baby size both larger and smaller than average, non-singleton birth, shorter maternal height, higher maternal BMI, and being enrolled in health insurance were all positively associated with higher odds of C-section. Living in an urban area and completing antenatal care more than four times were also significant predictors for C-section (S5 Table).

## Discussion

A few findings from our study deserve attention. First, although the prevalence of C-section among institutional delivery increased between 1999 and 2006, it declined between 2006 and 2016. Decomposing births by delivery place (home, public, and private institution) can provide insights into the observed trend. Based on NFHS II, III, and IV, there was a total of 10% point increase in institutional delivery between 1999 and 2006, with a similar margin of increase among public and private providers (4.2% and 5.6% respectively). However, an increase in institutional delivery between 2006 and 2016 was much greater, i.e. 32.8% point and was disproportionately concentrated in public institutions where C-section is less likely to be performed (33.3% point increase among public providers and 0.5% point decrease among private providers).

India launched Janani Suraksha Yojana (JSY) in 2005 to encourage institutional delivery, which had a maternity incentive scheme that entitled women who delivered in a government or accredited private health institution to a certain amount of money [40, 41]. Despite the policy supporting involvement of private health providers, it has been predominantly a public sector program and resulted in lower utilization of private institutions [40]. This may explain a decrease in C-section prevalence in India between 2006 and 2016 during the substantial increase in institutional delivery.

Second, private providers showed a strong positive association with C-section in all waves and across all states. The public-private disparity in C-section has been widely reported in previous studies in both LMICs and HICs [10, 12, 42, 43]. Private providers have a few motivations for C-section. Physicians’ defensive approaches to avoid negligence claims would be stronger in private providers because litigation damages their reputation, which can result in less revenue [44, 45]. Also, additional cases of surgery are directly linked to their income for private doctors while doctors in public institutions are usually paid by a fixed amount of salary [46]. On the other hand, the possibility should be considered that a large disparity between public and private institutions may reflect actual medical needs. Private institutions are better in the availability and readiness of emergency obstetric care than public institutions, especially at the lower-level [47]. Therefore, high-risk women may inevitably choose the private institution due to inadequate or lack of quality service in the public institution.

Third, stratified analyses revealed that the gap in C-section between public and private providers was bigger among less-educated and poorer women. This also could be related to the difference in the quality of service between public and private providers. India, like many other LMICs, suffers from a lack of obstetricians in the public sector, especially at the primary level. For example, among an estimated 2,000 obstetricians in Gujarat, three quarters work in the private sector, and even those working in the government sector are largely in medical college or in large district hospitals [48, 49]. Women with low SES are likely to use lower-level institutions such as clinics or small district hospitals rather than higher-level institutions due to lack of financial and geographical access. Because lower-level public institutions rarely have the proper equipment or professionals for surgery, women in an emergency may have no choice but to go to the private clinic either by their own decision or by transfer from other public clinics. On the other hand, women with higher SES are more likely to afford to use higher-level institutions which are usually equipped for C-section whether public or private.

Meanwhile, the tendency of private providers to encourage C-section cannot be ruled out as one of the mechanisms for a bigger gap in C-section between private and public providers among lower SES women [8, 14, 36, 50]. C-sections among wealthy and highly educated women is likely to be chosen by their own preference. Previous studies have reported that women opt for C-section to avoid labor pain [12], from misbeliefs that C-section is safer than vaginal delivery and can protect the baby’s brain [51], or to pick an auspicious date or time of birth [52]. These kinds of elective C-sections are luxurious choice only for affluent women because elective C-section is not covered by insurance and thus is paid out-of-pocket [53]. Highly educated women have a higher chance to obtain information on advanced delivery technique, especially about positive aspects. Besides, the decision-making power on the utilization of healthcare services is stronger among educated women [54]. Therefore, women who are wealthy or well-educated would demand C-section whether they deliver in private or public institutions. On the other hand, the choice of C-section among women with lower SES is more likely to be initiated by providers rather than their own preference. Poorly educated women are less likely to obtain accurate information [55, 56], and providers, especially private providers, may take advantage of this ignorance to induce demand for C-section. This might be a big concern because C-section is an economic burden on poor households [57, 58]. Mohanty et al. reported that the mean OOP expenditure of C-section in the private institutions was at least four times higher than that in the public institutions, and there was no significant difference in OOP expenditure of C-section in private institutions between better-off and poorer states [15]. Although some anecdotal evidence exists, it is not easy to demonstrate provider-induced demand with empirical evidence since there are many potential confounders uncontrollable in observational studies [59]. Therefore, careful monitoring of the C-section rate among private providers through diversified analysis would be required.

### Strength and limitations

This is the first study to examine the secular trend in the prevalence of C-section and its association with the type of provider among institutional delivery adjusted for a comprehensive set of variables based on nationally representative surveys. This study is also the first to demonstrate the different pattern in the association between type of provider and C-section by maternal education and wealth level.

On the other hand, this study has several limitations that need to be acknowledged. First, although we tried to control for potential confounders for the association between type of provider and C-section as comprehensively as the NFHS data allowed, there is a high chance that important factors representing medical risks were missing that were not collected in the NFHS. Therefore, special care should be taken to interpret the observed associations. Second, some clinical information such as the baby size, symptoms during delivery, and experience of miscarriage, abortion, or stillbirth was based on maternal self-report. This raises a concern that their responses might be based on subjective opinion or are subject to recall bias even if we restricted the study sample to the last birth to minimize it. Third, a few variables such as maternal BMI, smoking, and drinking were measured at the time of the survey, not during pregnancy, and hence there may be bias if changes in BMI and health behaviors for some women occurred non-differentially after delivery.

## Conclusion

The disparity in C-section between private and public providers has increased over the last 15 years and was higher among poorer and less-educated women. While further analyses are needed, our study suggests that the government needs to closely monitor the behavior of providers to ensure that C-section is performed only when medically necessary. Also, lower-level public institutions need to be properly equipped for emergency care so that women with low SES can use it without difficulty.

## Supporting information

S1 Fig Derivation process of the analytical sample. (DOCX)

S1 Table Comparison of model-fit between fully adjusted and parsimonious models. (DOCX)

S2 Table Descriptive statistics of analytic and original sample (%). (DOCX)

S3 Table Descriptive statistics of 36 states and 29 states after excluding 7 union territories. (DOCX)

S4 Table Stratified analyses by state based on NFHS IV (unit: OR). (DOCX)

S5 Table Determinants of C-section in 2016 (NFHS IV). (DOCX)
