# Supplementary figures and images for: Association between the type of provider and Cesarean section delivery in India: A socioeconomic analysis of the National Family Health Surveys 1999, 2006, 2016
Source: PLoS One. 2021 Mar 8;16(3):e0248283. doi: 10.1371/journal.pone.0248283 (PMC7939292; doi:10.1371/journal.pone.0248283)

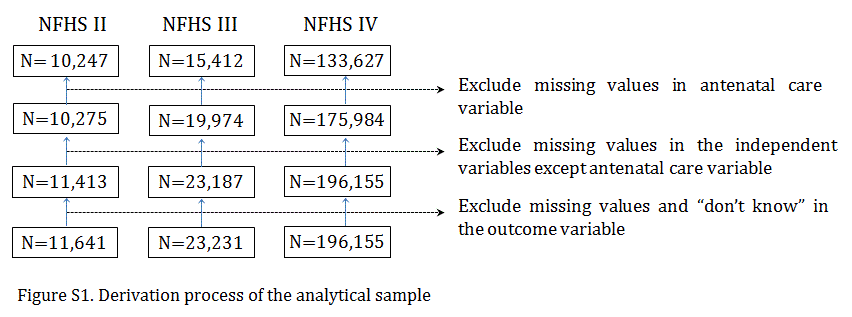

Supplement: S1 Fig — (DOCX) [file pone.0248283.s001.docx]
