# Supplementary material for: Association between the type of provider and Cesarean section delivery in India: A socioeconomic analysis of the National Family Health Surveys 1999, 2006, 2016
Source: PLoS One. 2021 Mar 8;16(3):e0248283. doi: 10.1371/journal.pone.0248283 (PMC7939292; doi:10.1371/journal.pone.0248283)
Supplement: S1 Table — (DOCX) [file pone.0248283.s002.docx]

S1 Table. Comparison of model-fit between fully adjusted and parsimonious models

| Year | Model | Odds ratio | | Pseudo LL | AIC | BIC | % Change | |
| --- | --- | --- | --- | --- | --- | --- | --- | --- |
|  |  | Private | private |  |  |  | AIC | BIC |
| 1999 | Fully adjusted | Ref | 1.39 | -4811.5 | 9672.9 | 9853.8 |  |  |
|  | Parsimonious^*^ | Ref | 1.40 | -4820.5 | 9688.9 | 9862.5 | 0.002 | 0.001 |
| 2006 | Fully adjusted | Ref | 1.59 | -7927.2 | 15901.6 | 16115.6 |  |  |
|  | Parsimonious^†^ | Ref | 1.59 | -7922.8 | 15910.4 | 16124.4 | 0.001 | 0.001 |
| 2016 | Fully adjusted | Ref | 3.72 | -54377.9 | 108825.7 | 109168.8 |  |  |
|  | Parsimonious ^∫^ | Ref | 3.72 | -54396.3 | 108854.6 | 109158.5 | 0.000 | 0.000 |

AIC: Akaike information criterion/ BIC: Bayesian information criterion /LL: Log Likelihood

* ^:^ A baby gender, smoking, drinking, and complication of pregnancy were excluded.

†: A baby gender, smoking, drinking, and urban were excluded.

∫: A baby gender, smoking, drinking, mother’s education, and urban were excluded.
