# Supplementary material for: Association between the type of provider and Cesarean section delivery in India: A socioeconomic analysis of the National Family Health Surveys 1999, 2006, 2016
Source: PLoS One. 2021 Mar 8;16(3):e0248283. doi: 10.1371/journal.pone.0248283 (PMC7939292; doi:10.1371/journal.pone.0248283)
Supplement: S2 Table — (DOCX) [file pone.0248283.s003.docx]

S2 Table. Descriptive statistics of analytic and original sample (%)

| **Variable** | NFHS II (1999) | | NFHS III (2006) | | NFHS IV (2016) | |
| --- | --- | --- | --- | --- | --- | --- |
|  | Analytic | Original | Analytic | Original | Analytic | Original |
| Age at pregnancy |  |  |  |  |  |  |
| <20 | 15.0 | 15.0 | 10.6 | 12.8 | 7.7 | 9.4 |
| ≧ 20 and <30 | 70.7 | 70.5 | 71.6 | 71.0 | 74.4 | 74.6 |
| ≧ 30 and <35 | 10.8 | 10.8 | 13.5 | 12.4 | 13.0 | 11.7 |
| ≧ 35 | 3.6 | 3.7 | 4.3 | 3.8 | 5.0 | 4.3 |
| Birth order |  |  |  |  |  |  |
| First | 43.8 | 43.2 | 39.4 | 45.0 | 36.8 | 43.0 |
| Second | 29.7 | 29.4 | 35.6 | 31.8 | 34.6 | 31.8 |
| Third | 14.0 | 14.2 | 13.8 | 12.5 | 15.9 | 14.5 |
| More than four | 12.5 | 13.3 | 11.2 | 10.7 | 12.7 | 11.8 |
| Gender of baby |  |  |  |  |  |  |
| Male | 52.9 | 53.4 | 54.6 | 53.1 | 54.6 | 52.5 |
| Female | 47.1 | 46.6 | 45.4 | 46.9 | 45.4 | 47.5 |
| Baby size |  |  |  |  |  |  |
| Very large | 0 | 0 | 4.3 | 4.2 | 5.6 | 5.5 |
| Larger than average | 17.9 | 17.4 | 21.5 | 21.2 | 13.0 | 12.6 |
| Average | 59.8 | 59.6 | 55.6 | 55.4 | 70.0 | 69.3 |
| Smaller than average | 18.1 | 28.4 | 13.5 | 13.4 | 8.7 | 8.9 |
| Very small | 4.2 | 4.5 | 5.1 | 5.7 | 2.6 | 2.9 |
| Plurality of pregnancy |  |  |  |  |  |  |
| Singleton | 98.6 | 98.2 | 98.9 | 97.9 | 99.1 | 98.2 |
| Twin or triplet | 1.5 | 1.8 | 1.1 | 2.1 | 0.9 | 1.8 |
| Short stature |  |  |  |  |  |  |
| Not short (height≧155cm) | 29.1 | 28.5 | 33.1 | 32.6 | 29.6 | 29.2 |
| Short (height<155cm) | 70.9 | 71.5 | 66.9 | 67.4 | 70.4 | 70.8 |
| BMI |  |  |  |  |  |  |
| >30 | 1.9 | 1.8 | 3.5 | 3.3 | 3.4 | 3.3 |
| ≧25 and <30 | 9.1 | 9.0 | 14.3 | 13.6 | 13.1 | 12.6 |
| ≧18 and <25 | 65.6 | 65.6 | 63.2 | 63.5 | 66.6 | 66.9 |
| <18 | 23.5 | 23.6 | 19.0 | 19.6 | 16.9 | 17.3 |
| Smoking |  |  |  |  |  |  |
| No | 99.0 | 99.0 | 91.2 | 91.3 | 91.6 | 91.4 |
| Yes | 1.0 | 1.0 | 8.8 | 8.7 | 8.4 | 8.5 |
| Alcohol |  |  |  |  |  |  |
| No | 98.8 | 98.9 | 98.4 | 98.3 | 98.4 | 98.5 |
| Yes | 1.2 | 1.1 | 1.6 | 1.7 | 1.6 | 1.5 |
| Complication |  |  |  |  |  |  |
| No | n.a | n.a | n.a | n.a | 47.3 | 47.6 |
| Yes | n.a | n.a | n.a | n.a | 52.7 | 52.4 |
| Terminated pregnancy |  |  |  |  |  |  |
| No | n.a | n.a | n.a | n.a | 89.1 | 89.5 |
| Yes | n.a | n.a | n.a | n.a | 10.9 | 10.5 |
| Maternal education |  |  |  |  |  |  |
| No education | 22.2 | 24.1 | 16.7 | 18.9 | 22.5 | 24.7 |
| Primary graduate or less | 16.5 | 16.4 | 12.0 | 12.2 | 12.8 | 13.5 |
| Secondary graduate or less | 40.3 | 39.1 | 54.0 | 53.2 | 51.4 | 50.2 |
| Collage or above | 21.0 | 20.5 | 17.4 | 15.7 | 13.3 | 11.6 |
| Type of residence |  |  |  |  |  |  |
| Urban | 49.5 | 48.9 | 57.7 | 56.8 | 28.5 | 27.1 |
| Rural | 50.5 | 51.1 | 42.3 | 43.2 | 71.5 | 72.9 |
| Caste |  |  |  |  |  |  |
| Scheduled caste | 14.3 | 14.4 | 15.2 | 16.1 | 19.5 | 20.1 |
| Scheduled tribe | 8.9 | 9.1 | 9.7 | 9.9 | 17.1 | 17.2 |
| Other backward class | 29.1 | 29.0 | 33.4 | 33.2 | 42.7 | 42.9 |
| Others | 47.7 | 47.5 | 41.7 | 40.8 | 20.7 | 19.7 |
| Wealth level |  |  |  |  |  |  |
| 1^st^ quintile(poorest) | 5.1 | 5.7 | 4.5 | 5.0 | 18.8 | 20.2 |
| 2^nd^ quintile | 8.9 | 9.5 | 8.7 | 9.5 | 21.1 | 22.2 |
| 3^rd^ quintile | 16.8 | 17.0 | 17.1 | 17.8 | 21.3 | 21.5 |
| 4^th^ quintile | 30.4 | 29.8 | 28.0 | 28.6 | 20.1 | 19.4 |
| 5^th^ quintile(richest) | 38.8 | 38.1 | 41.7 | 39.2 | 18.7 | 16.7 |
| Health Insurance |  |  |  |  |  |  |
| No | n.a | n.a | 92.8 | 95.7 | 84.7 | 85.6 |
| Yes | n.a | n.a | 7.3 | 4.3 | 15.3 | 14.4 |
| Place of delivery |  |  |  |  |  |  |
| Public | 54.6 | 54.3 | 51.7 | 52.1 | 70.6 | 71.9 |
| Private | 45.4 | 45.7 | 48.3 | 47.9 | 29.4 | 28.1 |
| Antenatal care more than 4 times |  |  |  |  |  |  |
| No | 34.6 | 36.6 | 26.5 | 26.4 | 45.6 | 45.3 |
| Yes | 65.4 | 63.4 | 73.5 | 73.6 | 54.4 | 54.7 |
