# Supplementary material for: Association between the type of provider and Cesarean section delivery in India: A socioeconomic analysis of the National Family Health Surveys 1999, 2006, 2016
Source: PLoS One. 2021 Mar 8;16(3):e0248283. doi: 10.1371/journal.pone.0248283 (PMC7939292; doi:10.1371/journal.pone.0248283)
Supplement: S3 Table — (DOCX) [file pone.0248283.s004.docx]

S3 Table. Descriptive statistics of 36 states and 29 states after excluding 7 union territories.

| **Variable** | N (%) | | | | Prevalence of C-sec | |
| --- | --- | --- | --- | --- | --- | --- |
|  | 36 states | | 29 states | | 36 states | 29 states |
| Age at pregnancy |  |  |  |  |  |  |
| <20 | 10,269 | (7.7) | 9,391 | (7.5) | 16.0 | 16.0 |
| ≧ 20 and <30 | 99,434 | (74.4) | 93,193 | (74.6) | 19.0 | 18.9 |
| ≧ 30 and <35 | 17,337 | (13.0) | 16,223 | (13.0) | 22.7 | 22.2 |
| ≧ 35 | 6,651 | (5.0) | 6,185 | (4.9) | 21.8 | 21.5 |
| Birth order |  |  |  |  |  |  |
| First | 16,977 | (12.7) | 16,092 | (12.9) | 6.5 | 6.5 |
| Second | 21,268 | (15.9) | 19,864 | (15.9) | 12.3 | 12.2 |
| Third | 46,258 | (34.6) | 43,190 | (34.6) | 20.9 | 20.7 |
| More than four | 49,188 | (36.8) | 45,846 | (36.7) | 25.5 | 25.5 |
| Gender of baby |  |  |  |  |  |  |
| Male | 73,008 | (54.6) | 68,398 | (54.7) | 19.3 | 19.2 |
| Female | 60,683 | (45.4) | 56,594 | (45.3) | 19.5 | 19.3 |
| Baby size |  |  |  |  |  |  |
| Very large | 7,481 | (5.6) | 7,150 | (5.7) | 24.9 | 24.7 |
| Larger than average | 17,434 | (13.0) | 16,339 | (13.1) | 22.8 | 22.6 |
| Average | 93,649 | (70.0) | 87,113 | (69.7) | 18.4 | 18.3 |
| Smaller than average | 11,671 | (8.7) | 11,079 | (8.9) | 18.8 | 18.4 |
| Very small | 3,456 | (2.6) | 3,311 | (2.6) | 19.7 | 19.4 |
| Plurality of pregnancy |  |  |  |  |  |  |
| Singleton | 132,542 | (99.1) | 123,937 | (99.2) | 19.2 | 19.1 |
| Twin or triplet | 1,149 | (0.9) | 1,055 | (0.8) | 38.7 | 38.1 |
| Mother’s height |  |  |  |  |  |  |
| Not short (height≧155cm) | 39,595 | (29.6) | 37,330 | (29.9) | 20.5 | 20.2 |
| Short (height<155cm) | 94,096 | (70.4) | 87,662 | (70.1) | 18.9 | 18.9 |
| BMI |  |  |  |  |  |  |
| >30 | 4,529 | (3.4) | 4,234 | (3.4) | 46.4 | 46.2 |
| ≧25 and <30 | 17,565 | (13.1) | 16,435 | (13.1) | 33.9 | 33.6 |
| ≧18.5 and <30 | 89,031 | (66.6) | 83,404 | (66.7) | 17.1 | 16.9 |
| <18.5 | 22,566 | (16.9) | 20,919 | (16.7) | 11.9 | 11.7 |
| Smoking |  |  |  |  |  |  |
| No | 122,513 | (91.6) | 114,176 | (91.3) | 19.9 | 19.8 |
| Yes | 11,178 | (8.4) | 10,816 | (8.7) | 13.6 | 13.6 |
| Alcohol |  |  |  |  |  |  |
| No | 131,579 | (98.4) | 123,027 | (98.4) | 19.5 | 19.3 |
| Yes | 2,112 | (1.6) | 1,965 | (1.6) | 15.3 | 15.8 |
| Maternal education |  |  |  |  |  |  |
| No education | 30,059 | (22.5) | 28,427 | (22.7) | 8.9 | 8.9 |
| Primary graduate or less | 17,130 | (12.8) | 16,336 | (13.1) | 13.0 | 13.0 |
| Secondary graduate or less | 68,746 | (51.4) | 63,966 | (51.2) | 21.3 | 21.3 |
| Collage or above | 17,756 | (13.3) | 16,263 | (13.0) | 36.1 | 35.8 |
| Type of residence |  |  |  |  |  |  |
| Urban | 38,138 | (28.5) | 35,401 | (28.3) | 28.6 | 28.4 |
| Rural | 95,553 | (71.5) | 89,591 | (71.7) | 15.8 | 15.6 |
| Caste |  |  |  |  |  |  |
| Scheduled caste | 26,098 | (19.5) | 25,038 | (20.0) | 16.9 | 16.8 |
| Scheduled tribe | 22,869 | (17.1) | 20,996 | (16.8) | 12.6 | 12.6 |
| Other backward class | 57,098 | (42.7) | 52,784 | (42.2) | 19.6 | 19.4 |
| Others | 27,626 | (20.7) | 26,174 | (20.9) | 27.0 | 26.7 |
| Wealth level |  |  |  |  |  |  |
| 1^st^ quintile(poorest) | 25,114 | (18.8) | 22,838 | (18.3) | 6.9 | 6.8 |
| 2^nd^ quintile | 28,206 | (21.1) | 26,726 | (21.4) | 11.7 | 11.6 |
| 3^rd^ quintile | 28,475 | (21.3) | 27,093 | (21.7) | 19.1 | 19.0 |
| 4^th^ quintile | 26,910 | (20.1) | 25,055 | (20.0) | 26.2 | 25.9 |
| 5^th^ quintile(richest) | 24,986 | (18.7) | 23,280 | (18.6) | 33.8 | 33.5 |
| Insurance |  |  |  |  |  |  |
| Covered | 20,466 | (15.3) | 19,243 | (15.4) | 22.5 | 22.1 |
| Not covered | 113,225 | (84.7) | 105,749 | (84.6) | 18.9 | 18.7 |
| Place of delivery |  |  |  |  |  |  |
| Public | 94,409 | (70.6) | 88,916 | (71.1) | 11.1 | 11.1 |
| Private | 39,282 | (29.4) | 36,076 | (28.9) | 39.4 | 39.3 |
| Antenatal care ≧ 4 times |  |  |  |  |  |  |
| No | 60,943 | (45.6) | 57,196 | (45.8) | 12.3 | 12.2 |
| Yes | 72,748 | (54.4) | 67,796 | (54.2) | 25.4 | 25.2 |
| Total | 133,691 | | 124,992 | |  |  |
| Prevalence |  | |  | | 19.3 | 19.3 |
