# Supplementary material for: Association between the type of provider and Cesarean section delivery in India: A socioeconomic analysis of the National Family Health Surveys 1999, 2006, 2016
Source: PLoS One. 2021 Mar 8;16(3):e0248283. doi: 10.1371/journal.pone.0248283 (PMC7939292; doi:10.1371/journal.pone.0248283)
Supplement: S4 Table — (DOCX) [file pone.0248283.s005.docx]

S4 Table. Stratified analyses by state based on NFHS IV (unit: OR)

| **States** | Public  (Ref) | Private | N | **States** | Public  (Ref) | Private | N |
| --- | --- | --- | --- | --- | --- | --- | --- |
| Andaman and nicobar Is | - | 9.28^**^ | 457 | Madhya pradesh | - | 5.84^***^ | 12,903 |
| Andhra pradesh | - | 3.50^***^ | 1,932 | Maharashtra | - | 2.22^***^ | 5,970 |
| Arunachal pradesh | - | 3.69^***^ | 1,706 | Manipur | - | 3.00^***^ | 2,746 |
| Assam | - | 4.41^***^ | 4,684 | Meghalaya | - | 3.57^***^ | 1,567 |
| Bihar | - | 13.31^***^ | 10,781 | Mizoram | - | 3.15^***^ | 2,710 |
| Chandigarh | - | 3.94^†^ | 127 | Nagaland | - | 2.42^***^ | 954 |
| Chhattisgarh | - | 11.05^***^ | 4,677 | Delhi | - | 2.28^***^ | 876 |
| Dadra and nagar haveli | - | 0.94 | 147 | Odisha | - | 5.43^***^ | 7,212 |
| Daman and diu | - | 6.03^***^ | 254 | Puducherry | - | 2.58^***^ | 861 |
| Goa | - | 2.67^**^ | 288 | Punjab | - | 2.52^***^ | 3,697 |
| Gujarat | - | 2.38^***^ | 4,623 | Rajasthan | - | 3.18^***^ | 9,681 |
| Haryana | - | 2.56^***^ | 4,609 | Sikkim | - | 2.62^**^ | 781 |
| Himachal pradesh | - | 4.14^***^ | 1,635 | Tamil nadu | - | 2.76^***^ | 5,885 |
| Jammu and kashmir | - | 1.88^***^ | 2,783 | Tripura | - | 5.42^***^ | 693 |
| Jharkhand | - | 9.70^***^ | 5,222 | Uttar pradesh | - | 6.41^***^ | 18,946 |
| Karnataka | - | 2.98^***^ | 4,708 | Uttarakhand | - | 4.47^***^ | 2,793 |
| Kerala | - | 1.41^**^ | 1,857 | West bengal | - | 7.29^***^ | 2,712 |
| Lakshadweep | - | 3.33^**^ | 206 | Telangana | - | 3.97^***^ | 1,461 |

1. Adjusted for age at pregnancy, birth order, baby gender, baby size, a plurality of pregnancy, short stature, BMI, smoking, drinking alcohol, history of delivery complication and terminated pregnancy, maternal education, type of residence, caste, wealth level, ANC more than 4 times, and health insurance status.
2. ^***^: p<0.001, ^**^ : p<0.01, ^*^ : p<0.05,^†^:p=0.05
3. N: Number of observations in analytic sample
