# Supplementary material for: Association between the type of provider and Cesarean section delivery in India: A socioeconomic analysis of the National Family Health Surveys 1999, 2006, 2016
Source: PLoS One. 2021 Mar 8;16(3):e0248283. doi: 10.1371/journal.pone.0248283 (PMC7939292; doi:10.1371/journal.pone.0248283)
Supplement: S5 Table — (DOCX) [file pone.0248283.s006.docx]

S5 Table. Determinants of C-section in 2016 (NFHS IV)

| **Variable** | | NFHS IV | | |
| --- | --- | --- | --- | --- |
|  |  | OR | 95% CI | |
| Demographic  factors | Age at pregnancy (ref=<20) |  |  |  |
|  | ≧ 20 and <30 | 1.28^***^ | 1.15 - | 1.42 |
|  | ≧ 30 and <35 | 2.02^***^ | 1.71 - | 2.39 |
|  | ≧ 35 | 2.62^***^ | 2.09 - | 3.30 |
|  | Birth order (ref=more than four) |  |  |  |
|  | Third | 1.85^***^ | 1.69 - | 2.03 |
|  | Second | 3.32^***^ | 2.94 - | 3.74 |
|  | First | 4.61^***^ | 4.14 - | 5.14 |
|  | Baby gender (ref=male) |  |  |  |
|  | Female | 1.00 | 0.97 - | 1.04 |
| Medical need  factors | Baby size (ref=average) |  |  |  |
|  | Very large | 1.37^***^ | 1.21 - | 1.54 |
|  | Larger than average | 1.20^***^ | 1.11 - | 1.30 |
|  | Smaller than average | 1.06 | 1.00 - | 1.12 |
|  | Very small | 1.15^*^ | 1.03 - | 1.28 |
|  | Plurality of pregnancy (ref=singleton) |  |  |  |
|  | Twin or triplet | 3.53^***^ | 3.03 - | 4.12 |
|  | Short stature (ref= not short(≧155cm)) |  |  |  |
|  | Short (<155cm) | 1.22^***^ | 1.14 - | 1.31 |
|  | BMI (ref=obese (BMI<18) |  |  |  |
|  | ≧18 and <25 | 1.25^***^ | 1.17 - | 1.34 |
|  | ≧25 and <30 | 2.20^***^ | 2.00 - | 2.41 |
|  | >30 | 3.26^***^ | 2.94 - | 3.62 |
|  | Smoking (ref=no) |  |  |  |
|  | Yes | 0.97 | 0.83 - | 1.14 |
|  | Alcohol (ref=no) |  |  |  |
|  | Yes | 1.16 | 0.80 - | 1.69 |
|  | Complication(ref=no) |  |  |  |
|  | Yes | 1.09 | 0.92 - | 1.30 |
|  | Terminated pregnancy (ref=no) |  |  |  |
|  | Yes | 1.11^*^ | 1.02 - | 1.22 |
| SES  factors | Maternal education (ref=none) |  |  |  |
|  | Primary graduate or less | 1.15^**^ | 1.03 - | 1.28 |
|  | Secondary graduate or less | 1.29^***^ | 1.13 - | 1.47 |
|  | Collage or above | 1.33^***^ | 1.13 - | 1.56 |
|  | Type or residency (ref=rural) |  |  |  |
|  | Urban | 1.15^***^ | 1.06 - | 1.24 |
|  | Caste (ref=scheduled tribe) |  |  |  |
|  | Scheduled caste | 1.32^*^ | 1.08 - | 1.60 |
|  | Other backward class | 1.18 | 0.99 - | 1.41 |
|  | Others | 1.34^***^ | 1.17 - | 1.53 |
|  | Wealth level (ref=1^st^ quintile (poorest)) |  |  |  |
|  | 2^nd^ quintile | 1.26^***^ | 1.17 - | 1.35 |
|  | 3^rd^ quintile | 1.62^***^ | 1.46 - | 1.80 |
|  | 4^th^ quintile | 1.69^***^ | 1.51 - | 1.90 |
|  | 5^th^ quintile(richest) | 1.53^***^ | 1.29 - | 1.80 |
| Institutional  factors | Insurance (ref=not covered) |  |  |  |
|  | Covered | 1.26^***^ | 1.07 - | 1.47 |
|  | Place of delivery (ref=public) |  |  |  |
|  | Private | 3.72^***^ | 2.93 - | 4.73 |
|  | ANC more than 4 times (ref=no) |  |  |  |
|  | Yes | 1.51^***^ | 1.35 - | 1.69 |
